# Daily Low-Volume Paracentesis and Clinical Complications in Patients With Refractory Ascites

**DOI:** 10.1001/jamanetworkopen.2023.22048

**Published:** 2023-07-06

**Authors:** Tammo L. Tergast, Marie Griemsmann, Lena Stockhoff, Kerstin Port, Benjamin Heidrich, Markus Cornberg, Heiner Wedemeyer, Henrike Lenzen, Nicolas Richter, Elmar Jaeckel, Benjamin Maasoumy

**Affiliations:** 1 Department of Gastroenterology, Hepatology, Infectious Diseases and Endocrinology, Hannover Medical School, Hannover, Germany; 2 German Centre for Infection Research, HepNet Study-House of the German Liver Foundation, Hannover, Germany; 3 Centre for Individualised Infection Medicine, Hannover, Germany; 4 Department of General, Visceral and Transplant Surgery, Hannover Medical School, Hannover, Germany; 5 Department for Liver Transplantation at University Health Network of the University of Toronto, Toronto, Canada

## Abstract

**Importance:**

The potential association of low-volume paracentesis of less than 5 L with complications in patients with ascites remains unclear, and individuals with cirrhosis and refractory ascites (RA) treated with devices like Alfapump or tunneled-intraperitoneal catheters perform daily low-volume drainage without albumin substitution. Studies indicate marked differences regarding the daily drainage volume between patients; however, it is currently unknown if this alters the clinical course.

**Objective:**

To determine whether the incidence of complications, such as hyponatremia or acute kidney injury (AKI), is associated with the daily drainage volume in patients with devices.

**Design, Setting, and Participants:**

This retrospective cohort study of patients with liver cirrhosis, RA, and a contraindication for a transjugular intrahepatic portosystemic shunt who received either device implantation or standard of care (SOC; ie, repeated large-volume paracentesis with albumin infusion), and were hospitalized between 2012 and 2020 were included. Data were analyzed from April to October 2022.

**Interventions:**

Daily ascites volume removed.

**Main outcomes and Measures:**

The primary end points were 90-day incidence of hyponatremia and AKI. Propensity score matching was performed to match and compare patients with devices and higher or lower drainage volumes to those who received SOC.

**Results:**

Overall, 250 patients with RA receiving either device implantation (179 [72%] patients; 125 [70%] male; 54 [30%] female; mean [SD] age, 59 [11] years) or SOC (71 [28%] patients; 41 [67%] male; 20 [33%] female; mean [SD] age, 54 [8]) were included in this study. A cutoff of 1.5 L/d or more was identified to estimate hyponatremia and AKI in the included patients with devices. Drainage of 1.5 L/d or more was associated with hyponatremia and AKI, even after adjusting for various confounders (hazard ratio [HR], 2.17 [95% CI, 1.24-3.78]; *P* = .006; HR, 1.43 [95% CI, 1.01-2.16]; *P* = .04, respectively). Moreover, patients with taps of 1.5 L/d or more and less than 1.5 L/d were matched with patients receiving SOC. Those with taps of 1.5 L/d or more had a higher risk of hyponatremia and AKI compared with those receiving SOC (HR, 1.67 [95% CI, 1.06-2.68]; *P* = .02 and HR, 1.51 [95% CI, 1.04-2.18]; *P* = .03), while patients with drainage of less than 1.5 L/d did not show an increased rate of complications compared with those receiving SOC.

**Conclusions and Relevance:**

In this cohort study, clinical complications in patients with RA performing low-volume drainage without albumin infusion were associated with the daily volume drained. Based on this analysis, physicians should be cautious in patients performing drainage of 1.5 L/d or more without albumin infusion.

## Key Points

**Question:**

Is the amount of ascites removed per day associated with the clinical outcome in patients with refractory ascites treated with tunneled-intraperitoneal catheters or Alfapump without albumin infusion?

**Findings:**

In this cohort study of 250 patients with refractory ascites, drainage of 1.5 L/d or more was associated with a higher incidence of hyponatremia or acute kidney injury than drainage of less than 1.5 L/d. Compared with the standard of care of repeated large volume paracentesis with albumin infusion, drainage of 1.5 L/d or more was also linked to more complications, while this was not the case in those with drainage less than 1.5 L/d.

**Meaning:**

These findings suggest that physicians should be cautious in patients with drainage of 1.5 L/d or more without albumin infusion.

## Teaser

This cohort study examines the association of daily drainage volume and the incidence of complications, such as hyponatremia or acute kidney injury, in patients with cirrhosis and refractory ascites treated with devices.

## Introduction

Liver cirrhosis is the result of chronic liver disease and associated with significant morbidity and mortality.^1^ Cirrhosis can be further divided into different stages according to the presence or absence of clinical significant portal hypertension and the occurrence of complications, such as variceal bleeding or ascites.^2^ Development of refractory ascites (RA) is a hallmark in end-stage liver cirrhosis, and treatment options are limited.^3,4^ When liver transplantation is not available, implantation of a transjugular-intrahepatic portosystemic shunt (TIPS) remains the therapy of choice. Unfortunately, there can be contraindications, such as recurrent hepatic encephalopathy or heart failure, that prohibit TIPS implantation in some patients.^5^ To date, repeated large-volume paracentesis (LVP) with albumin infusion is widely considered as standard of care (SOC) in this population that can be difficult to treat, but it is time-consuming, often poorly accepted by patients, and linked to an ongoing risk of paracentesis-associated complications, such as bleeding or infections.^3,4^

Recently, different devices like the Alfapump or tunneled-intraperitoneal catheters (PeCa) have been introduced to enable a home-based treatment for patients with RA. Studies showed that these devices decrease the need for paracentesis and can even improve quality of life.^6,7^ However, other studies report a higher incidence of adverse events, such as acute kidney injury (AKI) or hyponatremia.^6^ Hence, rehospitalization rates were increased compared with SOC.^8^ Besides presence of a device in the peritoneal cavity, daily low-volume taps without albumin substitution is the most eminent difference in contrast to patients receiving SOC. Prior studies^9,10^ demonstrated that LVP without albumin substitution leads to AKI or hyponatremia. However, if this is the case in those undergoing low-volume paracentesis (ie, less than 5 L per drain) remains a matter of debate.^4,11,12,13^

Importantly, patients using devices to perform daily low-volume drainage pose a completely new entity of patients with end-stage cirrhosis, and data about modifiable factors on the clinical course of this group are scarce. Previous studies documented large differences regarding the volume of ascites removed per day in patients with devices, ranging from 0.5 L/d to 2.5 L/d in some cases.^8,14,15^ Hence, the total volume drained over time differs drastically. It is currently unknown if this is associated with a different clinical course. This study investigated the association between daily low-volume drainage and clinical complications in patients with liver cirrhosis and RA with a particular focus on the amount of ascites removed per day.

## Methods

This cohort study was approved by the local ethics committee and followed the Declaration of Helsinki.^35^ All included patients have provided written informed consent for analysis of their data. This study followed the Strengthening the Reporting of Observational Studies in Epidemiology (STROBE) reporting guideline.

### Study Cohort

For this study, all patients with liver cirrhosis, RA, and a contraindication for TIPS who underwent at least 1 paracentesis and received either Alfapump or PeCa implantation between 2012 and 2020 at Hannover Medical School were considered. The diagnosis of RA was made according to current guidelines.^3,4^ Patients were excluded from analysis if they had a history of organ transplantation; had the presence any cancer disease, intraabdominal infection, or congenital immune dysfunction; were eligible for TIPS, and did not have sufficient evidence of cirrhosis. Details regarding placement of devices have been described previously.^8^ All patients received training regarding the handling of the implanted device and received a follow-up appointment 1 month after placement of the device in our liver outpatient unit to pull the stitches from the device. Follow up for patients with devices was started when the devices were on a stable daily drainage volume. In the follow-up, 13 patients changed their daily drainage volume and were censored to the respective time point. Regular albumin infusion was not performed in patients with devices. However, albumin administration was possible in case of other indications, such as spontaneous bacterial peritonitis (SBP), AKI, or hyponatremia, in concordance with current guidelines.^16^ After device implantation, the final daily drainage volume was set with the goal of a stable body weight. This was determined with regards to previous LVP frequency and body weight course after device implantation. This process took between 2 to 6 days for all included patients. No device had to be explanted during this period. To compare those with a device with patients who received SOC, only patients with RA and a contraindication for TIPS, who received SOC were considered for further analysis (eFigure 1 in Supplement 1). At our center it is routine clinical practice to offer a PeCa or Alfapump to patients with RA and present TIPS contraindications. However, some patients do not wish to have devices implanted and therefore receive repeated LVP and albumin infusion at our center. For this cohort, baseline was defined as the time of the first paracentesis at our center.

### Data Assessment

Study patients and laboratory values were extracted automatically from the clinical information system and afterwards carefully validated manually. Information regarding the daily amount of ascites drained or clinical complications were collected via patients’ medical records. Diagnosis of AKI and severe AKI (ie, AKI grade III) was made according to the criteria of the International Ascites Club.^17^

### Study Design

This was a retrospective cohort study. Primary end points of this study were 90-day incidence of hyponatremia (ie, serum sodium less than 130 mEq/L; to convert to mmol/L, multiply by 1), AKI, and severe AKI. Since hyponatremia is common in end-stage liver disease and often transient, patients with hyponatremia at baseline were followed from the day hyponatremia was resolved (ie, serum sodium greater than 135 mEq/L). Secondary end points were 90-day liver transplantation-free survival and rehospitalization within 90 days.

First, the potential association of the daily amount of ascites drained on the incidence of hyponatremia, AKI, and severe AKI was analyzed. The Youden-Index was used to identify an optimal cutoff to estimate occurrence of complications within 90 days (ie, hyponatremia, AKI, or severe AKI) (analysis I in eFigure 1 in Supplement 1). Next, the cohort was divided according to the cutoff and further analyzed (analysis 2 in eFigure 1 in Supplement 1). Afterwards, patients with either a high or low amount of ascites removed per day were matched via propensity score matching (PSM) with individuals who received SOC and compared regarding the previously mentioned end points (analysis 3 in eFigure 1 in Supplement 1).

### Statistical Analysis

All analyses were conducted using R version 4.2.1 (R Project for Statistical Computing), SPSS version 26 (IBM), and GraphPad Prism version 7 (GraphPad Software). Continuous data are presented as mean (SD) and were analyzed using either an unpaired *t* test or, if applicable, Mann-Whitney-*U* test or paired *t* test. Categorical data were analyzed using χ^2^ or McNemar testing, respectively. PSM was performed with nearest neighbor matching and a caliper of 0.25. Standardized mean differences were used to assess balance before and after matching (eTable 2 and 3 in Supplement 1). Time-to-event analysis was conducted with Fine and Gray’s competing risk analysis or log-rank testing. For competing risk analyses, either death or liver transplantation were considered as competing events. Schoenfeld residual plots were used to test whether the proportional hazard assumption was met in time-to-event analyses. Kaplan-Meier curves were used to illustrate survival. To adjust for potential confounding factors, multivariable competing risk analysis was conducted. Included factors were age, MELD score, platelet count, leukocyte count, and intake of nonselective β blockers (NSBB). The same parameters were used as matching factors for PPS-matching. All factors have previously been associated with survival in cirrhosis.^18,19,20^ *P* values ≤ .05 were considered statistically significant. Data were analyzed from April to October 2022.

## Results

Overall, 250 patients with RA were included in this study. Included patients received either device implantation (179 [72%] patients; 125 [70%] male; 54 [30%] female; mean [SD] age, 59 [11] years) or SOC (61 [28%]; 41 [67%] male; 20 [33%] female; mean [SD] age, 54 [8] years).

### Analysis 1: Association Between Volume Drainage and Clinical Complications

Of the 179 patients included in analysis 1, 152 (85%) received a PeCa, and 27 (15%) were treated with Alfapump. The mean (SD) removed volume per day was 1.64 (0.61) L. The total volume drained per day was significantly associated with 90-day risk of hyponatremia, AKI, and severe AKI (hyponatremia: hazard ratio [HR] per L/d, 1.92 [95% CI, 1.36-2.74]; *P* < .001; AKI: HR per L/d, 1.53 [95% CI, 1.14-2.03]; *P* = .004; severe AKI: HR per L/d, 1.99 [95% CI, 1.11-3.56]; *P* = .02), and these clinical complications increased with the volume of ascites removed per day (eFigure 2 in Supplement 1). Using the Youden-Index, a cutoff of 1.5 L/d was identified to estimate any complication within 90-day (sensitivity, 82%; specificity, 61%; area under the curve, 0.72) (eFigure 3 in Supplement 1). The cutoff of 1.5 L/d or more was used for further analysis.

### Analysis 2: Association Between Drainage of 1.5 L/d or Greater and Complications

Patients in the group with 1.5 L/d or more of drainage volume were older (mean [SD] age, 58 [10] years vs 62 [12] years; *P* = .03). Other baseline characteristics were comparable between both groups, such as MELD score, platelet count, or baseline serum albumin (Table 1).

**Table 1.** Baseline Characteristics of Patients According to the Daily Volume Drained

Characteristic	Patients, No. (%)		*P* value
	≥1.5 L/d Drained, n = 127	<1.5 L/d Drained, n = 52	
Sex			
Male	90 (71)	35 (67)	.58
Female	37 (29)	17 (33)	
Underlying liver disease			
Alcoholic cirrhosis	69 (54)	23 (45)	.27
Viral hepatitis	12 (9)	6 (12)	.77
NASH	14 (11)	7 (14)	.61
Other	32 (25)	15 (29)	.56
Contraindication for TIPS			
Risk for HE	72 (57)	21 (41)	.06
Heart failure	28 (22)	13 (25)	.62
Pulmonary hypertension	12 (9)	6 (12)	.64
Other	15 (11)	11 (22)	.10
Presence of varices	111 (87)	41 (80)	.35
History of variceal bleeding	22 (17)	7 (14)	.56
History of hyponatremia	65 (51)	24 (46)	.62
Alfapump	18 (14)	9 (18)	.71
MELD, mean (SD), points	17 (6)	17 (7)	.69
Creatinine, mean (SD), mg/dL	1.65 (0.76)	1.72 (0.90)	.64
Bilirubin, mean (SD), mg/dL	3.04 (2.69)	3.10 (2.10)	.93
INR, mean (SD)	1.44 (0.28)	1.39 (0.28)	.34
Leukocyte count, mean (SD), ×10^3^/μL	7.2 (4.4)	6.1 (5.1)	.18
Age, mean (SD), y	58 (10)	62 (12)	.03
Sodium, mean (SD), mEq/L	134 (6)	135 (6)	.19
ALT, mean (SD), IU/mL	34 (43)	34 (45)	.99
Albumin, mean (SD), g/L	29 (8)	30 (7)	.55
Platelet count, mean (SD), ×10^3^/μL	114 (73)	129 (116)	.26
History of SBP	70 (55)	27 (53)	.41
Rifaximin	57 (45)	22 (43)	.83
Diuretics	91 (72)	37 (73)	.90
NSBB	35 (28)	20 (39)	.13
PPI	109 (86)	43 (84)	.80

Abbreviations: ALT, alanine amino transferase; HE, hepatic encephalopathy; INR, international normalized ratio; NASH, nonalcoholic steatohepatitis; NSBB, nonselective β blockers; PPI, proton pump inhibitors; SBP, spontaneous bacterial peritonitis; TIPS, transjugular-intrahepatic portosystemic shunt.

SI conversion factors: To convert ALT to μkat/L, multiply by 0.0167; bilirubin to μmol/L, multiply by 17.104; creatinine to μmol/L, multiply by 88.4; platelet count to ×10^9^/L, multiply by 1; sodium to mmol/L, multiply by 1; white blood cell count to ×10^9^/L, multiply by 0.001.

**Figure 1.**
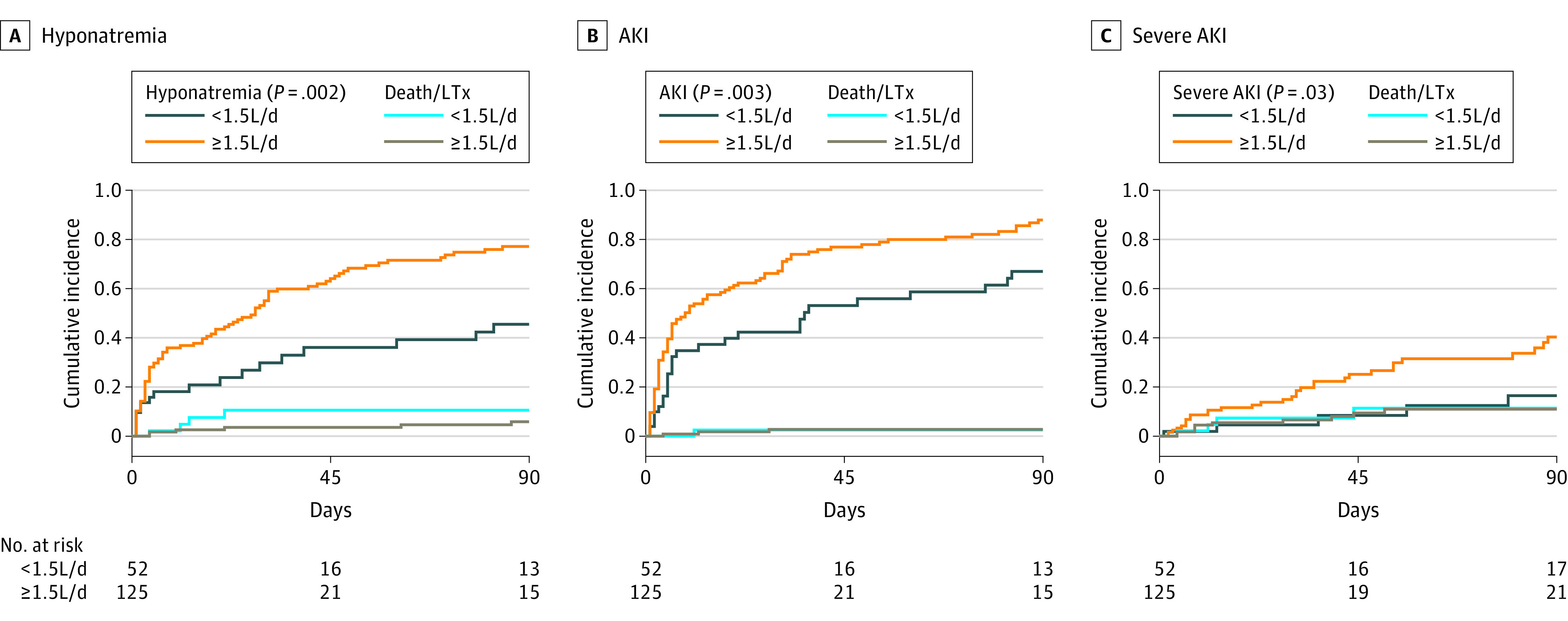
Ninety-Day Incidence of Hyponatremia, Acute Kidney Injury (AKI), and Severe AKI in Patients With Daily Paracentesis of 1.5 L/d or More and Less Than 1.5 L/d Death and liver transplantation were considered as competing events. Patients chronically dependent on hemodialysis at baseline were excluded from analysis regarding AKI or severe AKI. Overall, 13 patients adapted their daily drainage volume after a mean of 35 days in the follow-up (n = 9 in the 1.5 L/d or more group and n = 4 in the less than 1.5 L/d group). Schoenfeld residual plots indicated that the proportional hazards assumption was met in the respective analyses.

Within 90 days, risk of hyponatremia was significantly higher in those with drainage volume of 1.5 L/d or more (univariate HR, 2.26 [95% CI, 1.35-3.78]; *P* = .002) (Figure 1). The multivariable analysis revealed an independent association of drainage 1.5 L/d or more with the development of hyponatremia (HR, 2.17 [95% CI, 1.24-3.78]; *P* = .006) (eTable 1 in Supplement 1). Taps of 1.5 L/d or more were still associated with hyponatremia, when baseline sodium was added to the multivariable model (HR per L/d, 2.11 [95% CI, 1.26-3.65]; *P* = .009). Furthermore, the 90-day AKI risk was also higher in patients who had daily drainage of 1.5 L/d or more (univariate HR, 1.87 [95% CI, 1.24-2.82]; *P* = .003) (Figure 1). When adjusted for potential confounding factors, drainage volume of 1.5 L/d or more and MELD score were the only factors independently associated with AKI (1.5 L/d or more: HR, 1.43 [95% CI, 1.01-2.16]; *P* = .04; MELD score: HR, 1.05; [95% CI, 1.02-1.08]; *P* = .001) (eTable 1 in Supplement 1). Moreover, drainage of 1.5 L/d or more was associated with severe AKI (univariate HR, 2.84 [95% CI, 1.12-7.19]; *P* = .03) (Figure 1). The association remained statistically significant after adjusting for confounding factors (HR, 2.65; [95% CI, 1.21-6.54]; *P* = .03) (eTable 1 in Supplement 1). No association was found between patients with a drainage volume of 1.5 L/d or more and liver transplantation-free survival or the rehospitalization rate (eFigure 4 in Supplement 1).

Patients with drainage of 1.5 L/d or more showed decreasing mean (SD) serum sodium and albumin values in the follow-up, while their mean (SD) leucocyte count and C-reactive protein values increased (serum sodium: baseline, 134 mEq/L vs day 28: 129 mEq/L, *P* = .001; serum albumin: baseline, 2.90 g/dL vs day 28, 2.60 g/dL [to convert to g/L, multiply by 10]; *P* = .05; leucocyte count: baseline, 7.2 ×10^3^/μL vs day 28, 8.4 ×10^3^/μL [to convert to ×10^9^/L, multiply by 0.001]; *P* = .02; C-reactive protein: baseline, 22 mg/dL vs day 28, 32 mg/dL [to convert to mg/L, multiply; *P* = .007) (eFigure 5 in Supplement 1). In contrast, patients with paracentesis of less than 1.5 L/d of drainage showed stable values for C-reactive protein at baseline compared with day 28 (baseline, 18 mg/L vs day 28, 21 mg/dL; *P* = .007) (eFigure 5 in Supplement 1).

Furthermore, all patients with Alfapump were analyzed separately. Drainage volume of 1.5 L/d or more was associated with hyponatremia and AKI within 90-day (HR, 5.29 [95% CI, 1.24-22.11]; *P* = .02; HR, 3.36 [95% CI, 1.03-10.95]; *P* = .045, respectively). There was no association of severe AKI (eFigure 6 in Supplement 1). In patients with PeCa, taps of 1.5 L/d or more were significantly associated with hyponatremia, AKI, and severe AKI (HR, 1.97 [95% CI, 1.14-3.39]; *P* = .01; HR, 1.69 [95% CI, 1.69-2.61]; *P* = .02; HR, 2.56 [95% CI, 1.01-6.47]; *P* = .04, respectively) (eFigure 6 in Supplement 1).

### Analysis 3: Complications Among Patients With Devices vs Those Receiving SOC

**Figure 2.**
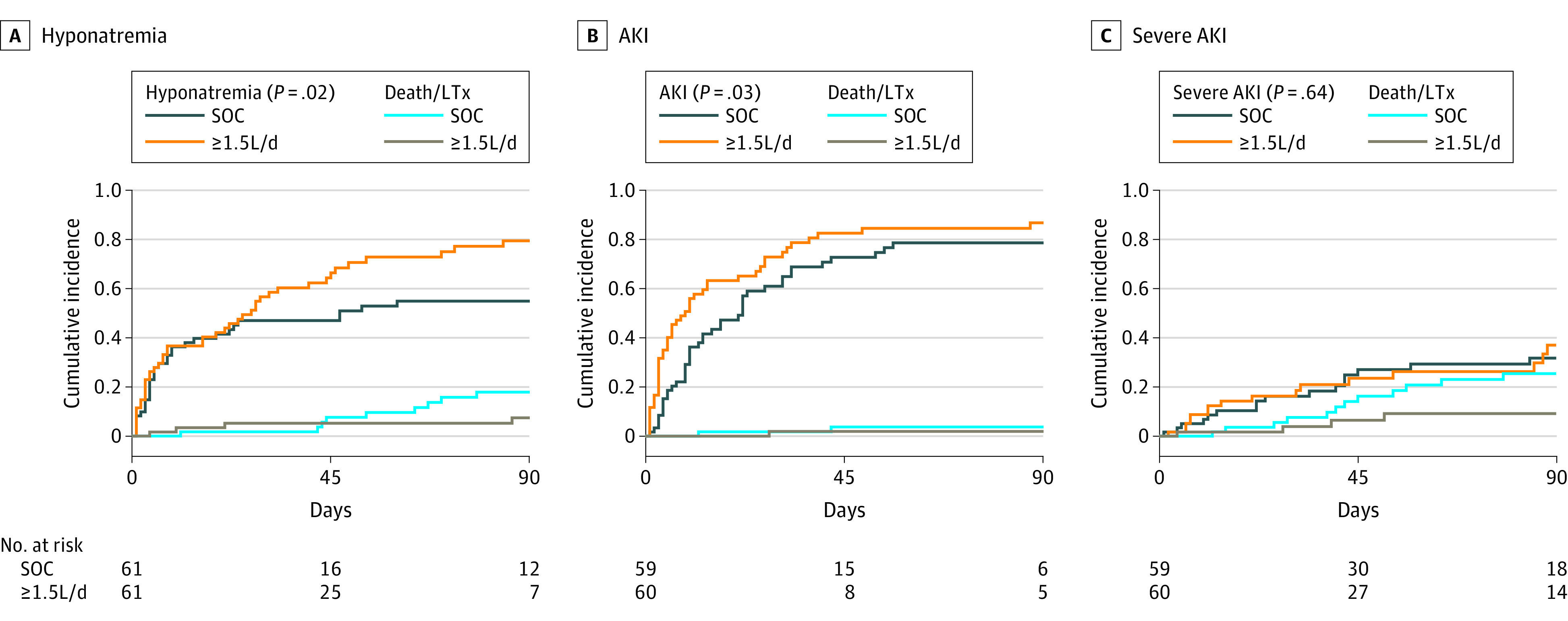
Ninety-Day Incidence of Hyponatremia, Acute Kidney Injury (AKI), and Severe AKI in Patients With Daily Paracentesis of 1.5 L/d or More and Standard of Care (SOC) Death and LTx were considered as competing events. Patients chronically dependent on hemodialysis at baseline were excluded from analysis regarding AKI/severe AKI. Schoenfeld residual plots indicated that the proportional hazards assumption was met in the respective analyses.

All patients with daily paracentesis of 1.5 L/d or more were matched with the SOC cohort (Table 2 and eTable 2 in Supplement 1). Patients in the group with drainage of 1.5 L/d or more received significantly less albumin per liter ascites drained within 90 days compared with the SOC cohort (5.9 g/L vs 12.6 g/L; *P* < .001). Compared to SOC, the 90-day risk of hyponatremia and AKI was higher in the group with drainage of 1.5 L/d or more (HR, 1.67 [95% CI, 1.06-2.68]; *P* = .02; HR, 1.51 [95% CI, 1.04-2.18]; *P* = .03, respectively), while severe AKI was not associated (HR, 1.18 [95% CI, 0.59-2.35]; *P* = .64) (Figure 2). The rehospitalization rate was higher in patients with devices who performed paracentesis of 1.5 L/d or more (HR, 2.27 [95% CI, 1.34-3.82]; *P* = .002) (eFigure 7 in Supplement 1).

**Table 2.** Baseline Characteristics

Characteristic	Patient, No. (%), (n = 61)		*P* value	Patient, No. (%), (n = 31)		*P* value
	Daily paracentesis ≥1.5 L/d	SOC		Daily paracentesis <1.5 L/d	SOC	
Sex						
Male	39 (64)	41 (67)	.85	20 (65)	21 (68)	.89
Female	22 (36)	20 (33)		11 (35)	10 (32)	
Underlying liver disease						
Alcoholic cirrhosis	39 (64)	32 (52)	.21	10 (32)	18 (58)	.06
Viral hepatitis	7 (12)	12 (20)	.09	5 (16)	4 (13)	>.99
NASH	5 (8)	3 (5)	.73	3 (10)	1 (3)	.63
Other	10 (16)	16 (26)	.15	13 (42)	8 (26)	.19
Contraindication for TIPS						
Risk for HE	40 (66)	45 (74)	.46	13 (42)	19 (61)	.17
Heart failure	6 (10)	8 (13)	.79	8 (26)	7 (23)	>.99
Pulmonary hypertension	4 (7)	4 (7)	>.99	5 (16)	5 (16)	>.99
Other	11 (18)	4 (7)	.22	5 (16)	1 (3)	.33
Presence of varices	46 (75)	44 (72)	.69	23 (74)	22 (71)	.89
History of variceal bleeding	10 (16)	11 (18)	.77	7 (23)	6 (19)	.68
History of hyponatremia	31 (51)	32 (52)	.94	15 (48)	13 (42)	.75
MELD, mean (SD), points	16 (5)	17 (5)	.19	17 (7)	16 (5)	.52
Creatinine, mean (SD), mg/dL	1.52 (0.69)	1.71 (0.93)	.36	1.74 (1.05)	140 (92)	.56
Bilirubin, mean (SD), mg/dL	2.34 (2.16)	2.98 (2.81)	.18	2.51 (2.51)	2.81 (2.98)	.65
INR, mean (SD)	1.41 (0.26)	1.49 (0.33)	.44	1.41 (0.28)	1.43 (0.22)	.67
Leukocyte count, mean (SD), ×10^3^/μL	8.0 (5.3)	8.2 (4.4)	.84	6.3 (6.5)	7.3 (3.8)	.27
Age, mean (SD), y	61 (9)	61 (7)	.83	56 (11)	57 (9)	.90
Sodium, mean (SD), mmol/L	133 (6)	133 (6)	.51	135 (5)	134 (4)	.72
ALT, mean (SD), IU/mL	27 (22)	34 (52)	.29	43 (59)	32 (10)	.08
Albumin, mean (SD), g/L	27 (6)	27 (5)	.34	30 (6)	29 (5)	.20
Platelet count, mean (SD), ×10^3^/μL	120 (85	131 (89)	.30	126 (98)	154 (119)	.38
History of SBP	28 (46)	22 (36)	.21	10 (32)	12 (39)	.55
Rifaximin	29 (48)	27 (44)	.92	14 (45)	14 (45)	>.99
Diuretics	48 (79)	46 (75)	.82	26 (84)	25 (81)	>.99
NSBB	21 (34)	26 (43)	.33	14 (45)	14 (45)	>.99
PPI	49 (80)	48 (79)	.89	25 (81)	23 (74)	.88

Abbreviations: ALT, alanine amino transferase; HE, hepatic encephalopathy; INR, international normalized ratio; MELD, model for end-stage liver disease; NASH, nonalcoholic steatohepatitis; NSBB, nonselective β blockers; PPI, proton pump inhibitors; SBP, spontaneous bacterial peritonitis; SOC, standard of care; TIPS, transjugular-intrahepatic portosystemic shunt.

SI conversion factors: To convert ALT to μkat/L, multiply by 0.0167; bilirubin to μmol/L, multiply by 17.104; creatinine to μmol/L, multiply by 88.4; platelet count to ×10^9^/L, multiply by 1; sodium to mmol/L, multiply by 1; white blood cell count to ×10^9^/L, multiply by 0.001.

**Figure 3.**
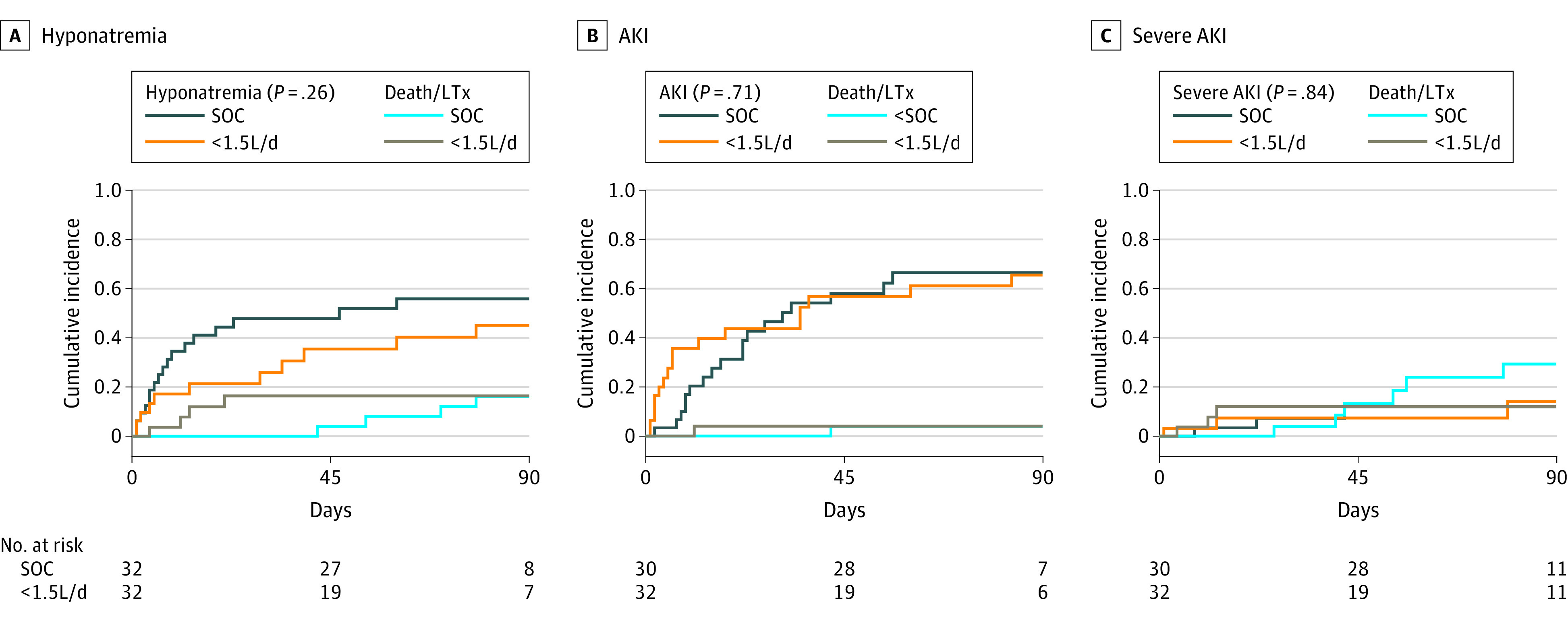
Ninety-Day Incidence of Hyponatremia, Acute Kidney Injury (AKI), and Severe AKI in Patients With Daily Paracentesis Less Than 1.5 L/d A, Hyponatremia; B, AKI; and C, severe AKI in patients with daily paracentesis of less than 1.5 L/d and SOC. Death and liver transplantation were considered as competing events. Patients chronically dependent on hemodialysis at baseline were excluded from analysis regarding AKI or severe AKI. Schoenfeld residual plots indicated that the proportional hazards assumption was met in the respective analyses. SOC indicates standard of care.

Last, those with daily drainage of less than 1.5 L/d underwent matching with patients who received SOC (Table 2 and eTable 3 in Supplement 1). The mean (SD) albumin infusion per liter ascites drained was significantly higher in the SOC cohort (2.8 g/L vs 13.9 g/L; *P* < .001) and 90-day risk of hyponatremia, AKI, and severe AKI was not associated (HR, 0.66 [95% CI, 0.32-1.37]; *P* = .26; HR, 1.13 [95% CI, 0.59-2.19]; *P* = .71; HR, 1.17 [95% CI, 0.25-5.4]; *P* = .84, respectively) (Figure 3). Additionally, 90-day rehospitalization was not associated with either group (HR, 1.83; [95% CI, 0.78-4.28]; *P* = .16) (eFigure 8 in Supplement 1).

## Discussion

In this study, we investigated the long-term association between ascites volume drained per day and clinical outcome in patients treated with devices. Higher quantities of ascites removed per day were associated with AKI and hyponatremia. Compared with patients receiving SOC, those with daily taps of 1.5 L/d or more showed higher rates of AKI, hyponatremia, and rehospitalization, while this was not the case in individuals with daily drainage of less than 1.5 L/d.

Patients with end-stage liver disease, RA, and contraindications for TIPS are difficult to treat and have frequent complications.^13,21^ To enable a more self-determined life and to save costs from repeated LVP in combination with albumin infusion, devices like PeCa or Alfapump have been introduced.^22^ However, little is known about the association of daily low-volume ascites taps, and this distinct group represents a new entity of patients. Previous studies^6,8,23^ indicated higher rates of kidney impairment or rehospitalization, while others did not find such an association in those treated with devices compared with SOC. In addition, the potential effect of taps with volumes less than 5 L without albumin infusion is currently insufficiently studied. Of note, Macken et al^14^ investigated a small cohort of patients with PeCa, who drained a limited volume of up to 6 L per week and did not observe higher rates of kidney failure and hyponatremia compared with SOC. Hence, daily drainage of low volumes (ie, drainage of less than 1 to 1.5 L/d) may not lead to more complications than SOC.

Besides the increased incidence of clinical end points in patients with drainage of 1.5 L/d or more, we observed a significant decrease of serum sodium while leucocyte count and C-reactive protein increased in patients with taps of 1.5 L/d or more after 28 days, similar to patients undergoing LVP without albumin infusion.^10,24^ Interestingly, this was not the case in patients with daily drainage of 1.5 L/d or less. In the setting of LVP without albumin substitution, postparacentesis circulatory dysfunction (PPCD) occurs frequently and is most likely the result of effective blood volume reduction.^13^ PPCD is associated with AKI, hyponatremia, and decreased survival.^25^ Repeated low-volume paracentesis of 1.5 L/d or more might have led to a PPCD-like state over time and could explain the clinical observations in our cohort. Data about the potential associations of repeated low-volume drainage regarding an altered clinical course and the occurrence of PPCD are scarce, and it has been suggested that removal of less than 5 L ascites per drain does rarely lead to PPCD,^12,26^ at least in the setting of a single paracentesis. Thus, albumin infusion in these patients remains a matter of debate.^4^ However, PPCD has already been reported in patients with Alfapump, and our data indicate that complications occur with increasing drainage volumes, even in the setting of low-volume paracentesis.^15^ In our cohort, none of the patients received regular albumin infusion after drainage and albumin was only administered for other indications like SBP, AKI, or hyponatremia. Strikingly, albumin infusion can effectively prevent PPCD and is recommended after paracentesis of more than 5 L.^3,27,28^ Thus, it is tempting to estimate that albumin infusion could decrease complications in individuals with daily drains of 1.5 L/d or more. Treatment could be adapted in those who are in need of taps of 1.5 L/d or more. One could only drain less than 1.5 L/d and perform LVP every few weeks (via the device) with albumin infusion in an outpatient unit. However, this would contradict the idea of home-based therapy. Another potential option could be drainage of 1.5 L/d or more and infusion of albumin with the help of a dedicated care service, in the same manner as palliative patients receive infusions in a home-based care. Future studies might therefore investigate the association of albumin infusions, especially in those with higher daily drainage volumes.

Patients with RA frequently have sarcopenia and malnutrition. Some of these cirrhosis-related states often act in concert of a vicious circle of catabolism.^29^ Ascites is the result of selective ultrafiltration, and it contains electrolytes, nutrients, or small proteins like albumin.^30^ Hence, daily drainage of larger ascites quantities may have further amplified the preexisting catabolic state. Moreover, occurrence hyponatremia was associated with an increased daily drainage volume. In contrast to the well-defined hypervolemic dilutional hyponatremia which can occur as a result of peripheral vasodilatation, kidney hypoperfusion, and increased activation of the renin-angiotensin-aldosterone system,^31^ desalination could also be the result of daily taps. Salt restriction of 2 to 3 g/d is recommended in individuals with ascites.^3,4^ However, device-associated desalination has been described in patients with malignant neoplasm–related ascites and patients present with (intravasal) hypovolemic hyponatremia.^32,33^ Since ascites and serum contain similar amounts of sodium, drainage of 1.5 L/d or more leads to a sodium loss of more than 4.5 g/d, if the serum sodium is higher than 135 mmol/L.^8,34^ Hence, sodium restriction over time could be deleterious in these patients.

### Limitations

This study has limitations. First, the retrospective design could have led to undetected group differences between individuals with different amounts of ascites drained. Furthermore, a higher ascites production could be an indicator for an even more advanced state of cirrhosis in the setting of RA. However, important baseline characteristics were comparable between both groups, and we adjusted for important risk factors via multivariable analysis. Additionally, we used a composite endpoint to determine the cutoff. The optimal cutoff to estimate Hyponatremia was 1.45 L/d and the optimal cutoff to estimate AKI was 1.6 L/d. However, since a cutoff of 1.5 L/d is easier to apply and near the optimal cutoff for AKI and hyponatremia we decided to use a composite endpoint. Also, we only had 27 patients with an Alfapump in our cohort, hence information regarding the association of the device type is limited, even if the clinical outcomes of a higher drainage volume on the clinical course were observed in both device groups. Moreover, nonrandomization between patients with devices and SOC prohibits final conclusion regarding clinical end points. Nonetheless, we adjusted for this via PSM to enable comparability between both groups.

## Conclusions

In conclusion, our data indicate that the risk of clinical complications in those performing repeated low-volume paracentesis without albumin substitution is associated with the volume drained per day. Drainage of 1.5 L/d or more associates with a higher risk for hyponatremia, AKI, and increasing inflammation while those with drainage of less than 1.5 L/d show comparable complication rates with those receiving SOC.

## Notes

Supplement 1. **eFigure 1.** Study Design **eFigure 2. **Ninety-Day Incidence of Hyponatremia, AKI, and Severe AKI **eFigure 3. **ROC Curve **eFigure 4.** Ninety-Day Liver Transplantation-Free Survival and Rehospitalization Rate **eFigure 5.** Serum Sodium, Serum Albumin Values, and Leukocyte Count Within 28-Day Follow Up **eFigure 6.** Ninety-Day Incidences of Hyponatremia, AKI, and Severe AKI in Patients With Alfapump or PeCa **eFigure 7.** Rehospitalization Rate in Patients With Either SOC or Daily Drainage of 1.5 L or More **eFigure 8.** Rehospitalization Rate in Patients With Either SOC or Daily Drainage of Less Than 1.5 L **eTable 1.** Results of Multivariable Competing Risk Analysis **eTable 2.** Standardized Mean Differences Before and After PSM in Patients With Daily Drainage of 1.5 L/d or More Compared With Patients With SOC **eTable 3.** Standardized Mean Differences Before and After PSM in Patients With Daily Drainage of Less Than 1.5 L/d Compared With Patients With SOC

Supplement 2.
Data Sharing Statement
