# Supplementary material for: Daily Low-Volume Paracentesis and Clinical Complications in Patients With Refractory Ascites
Source: JAMA Netw Open. 2023 Jul 6;6(7):e2322048. doi: 10.1001/jamanetworkopen.2023.22048 (PMC10326647; doi:10.1001/jamanetworkopen.2023.22048)
Supplement: Supplement 1. — eFigure 1. Study Design eFigure 2. Ninety-Day Incidence of Hyponatremia, AKI, and Severe AKI eFigure 3. ROC Curve eFigure 4. Ninety-Day Liver Transplantation-Free Survival and Rehospitalization Rate eFigure 5. Serum Sodium, Serum Albumin Values, and Leukocyte Count Within 28-Day Follow Up eFigure 6. Ninety-Day Incidences of Hyponatremia, AKI, and Severe AKI in Patients With Alfapump or PeCa eFigure 7. Rehospitalization Rate in Patients With Either SOC or Daily Drainage of 1.5 L or More eFigure 8. Rehospitalization Rate in Patients With Either SOC or Daily Drainage of Less Than 1.5 L eTable 1. Results of Multivariable Competing Risk Analysis eTable 2. Standardized Mean Differences Before and After PSM in Patients With Daily Drainage of 1.5 L/d or More Compared With Patients With SOC eTable 3. Standardized Mean Differences Before and After PSM in Patients With Daily Drainage of Less Than 1.5 L/d Compared With Patients With SOC [file jamanetwopen-e2322048-s001.pdf]

Supplemental Online Content

Tergast TL, Griemsmann M, Stockhoff L, et al. Daily low-volume paracentesis and clinical complications in patients with refractory ascites. *JAMA Netw Open*. 2023;6(7):e2322048. doi:10.1001/jamanetworkopen.2023.22048

eFigure 1. Study Design
eFigure 2. Ninety-Day Incidence of Hyponatremia, AKI, and Severe AKI
eFigure 3. ROC Curve
eFigure 4. Ninety-Day Liver Transplantation Free Survival and Rehospitalization Rate
eFigure 5. Serum Sodium, Serum Albumin Values, and Leukocyte Count Within 28-Day Follow Up
eFigure 6. Ninety-Day Incidences of Hyponatremia, AKI, and Severe AKI in Patients With Alfapump or PECA
eFigure 7. Rehospitalization Rate in Patients With Either SOC or Daily Drainage of 1.5 L or More
eFigure 8. Rehospitalization Rate in Patients With Either SOC or Daily Drainage of Less Than 1.5 L
eTable 1. Results of Multivariable Competing Risk Analysis
eTable 2. Standardized Mean Differences Before and After PSM in Patients With Daily Drainage of 1.5 L/d or More Compared With Patients With SOC
eTable 3. Standardized Mean Differences Before and After PSM in Patients With Daily Drainage of Less Than 1.5 L/d Compared With Patients With SOC

This supplemental material has been provided by the authors to give readers additional information about their work.

© 2023 Tergast TL et al. *JAMA Network Open.*

eFigure 1. Study Design

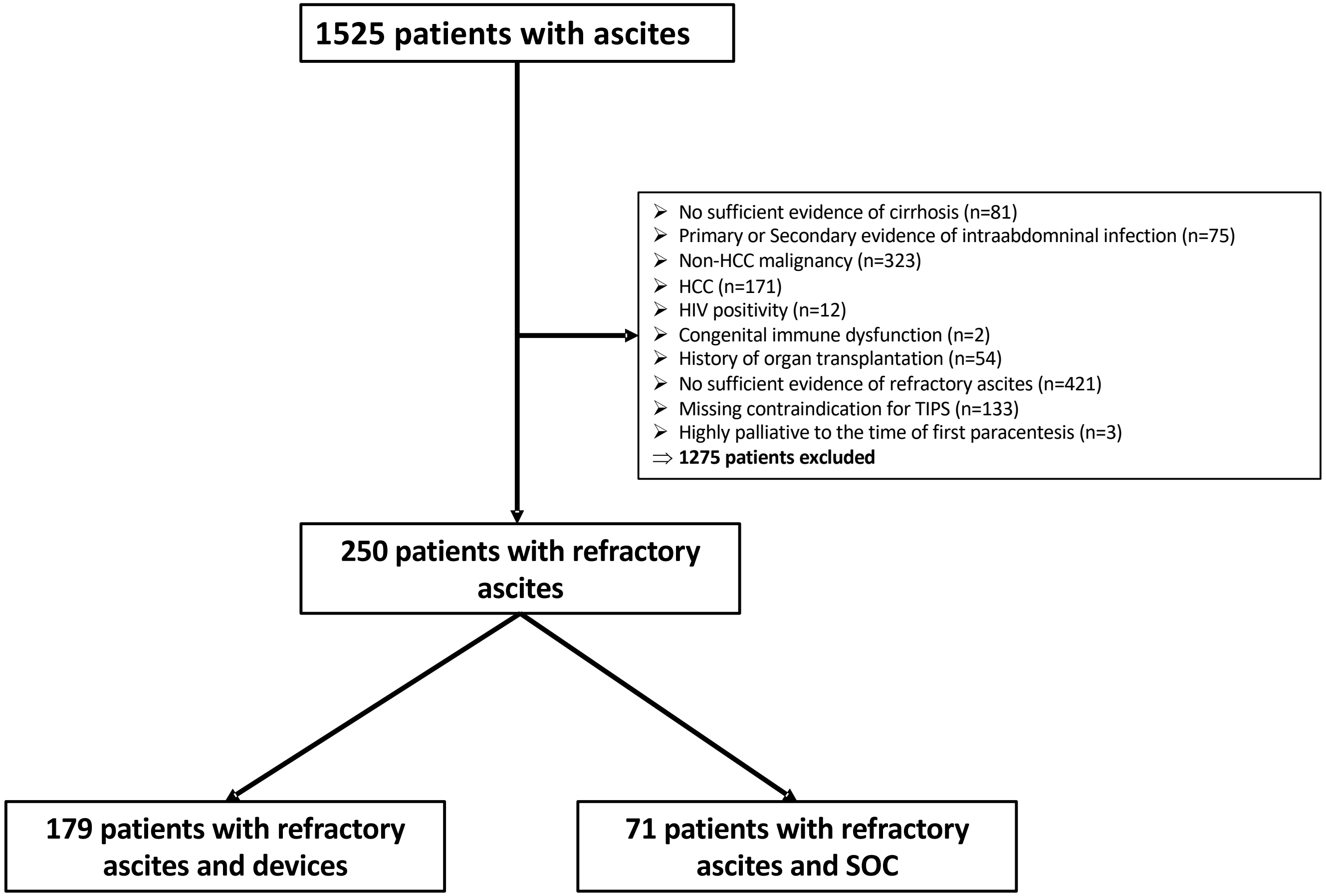

In- and exclusion criteria. All patients without TIPS contraindication ultimately underwent TIPS implantation. Three patients were excluded due to being highly palliative to the time of first paracentesis. They were ultimately excluded from the SOC cohort since they only received symptomatic treatment and did not receive albumin. All three patients died within 48 hours after initial admission.

© 2023 Tergast TL et al. *JAMA Network Open.*

eFigure 1. Study Design

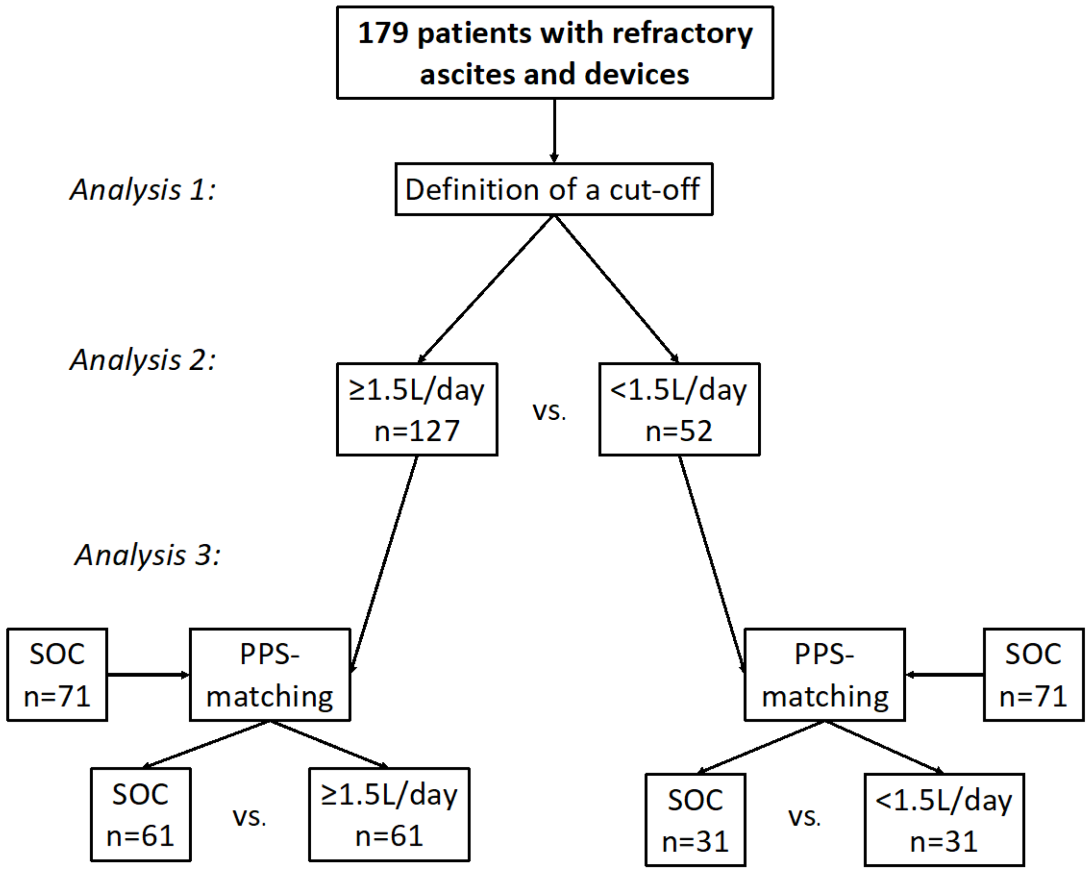

© 2023 Tergast TL et al. *JAMA Network Open.*

eFigure 2. 90-Day Incidence of Hyponatremia, AKI, and Severe AKI

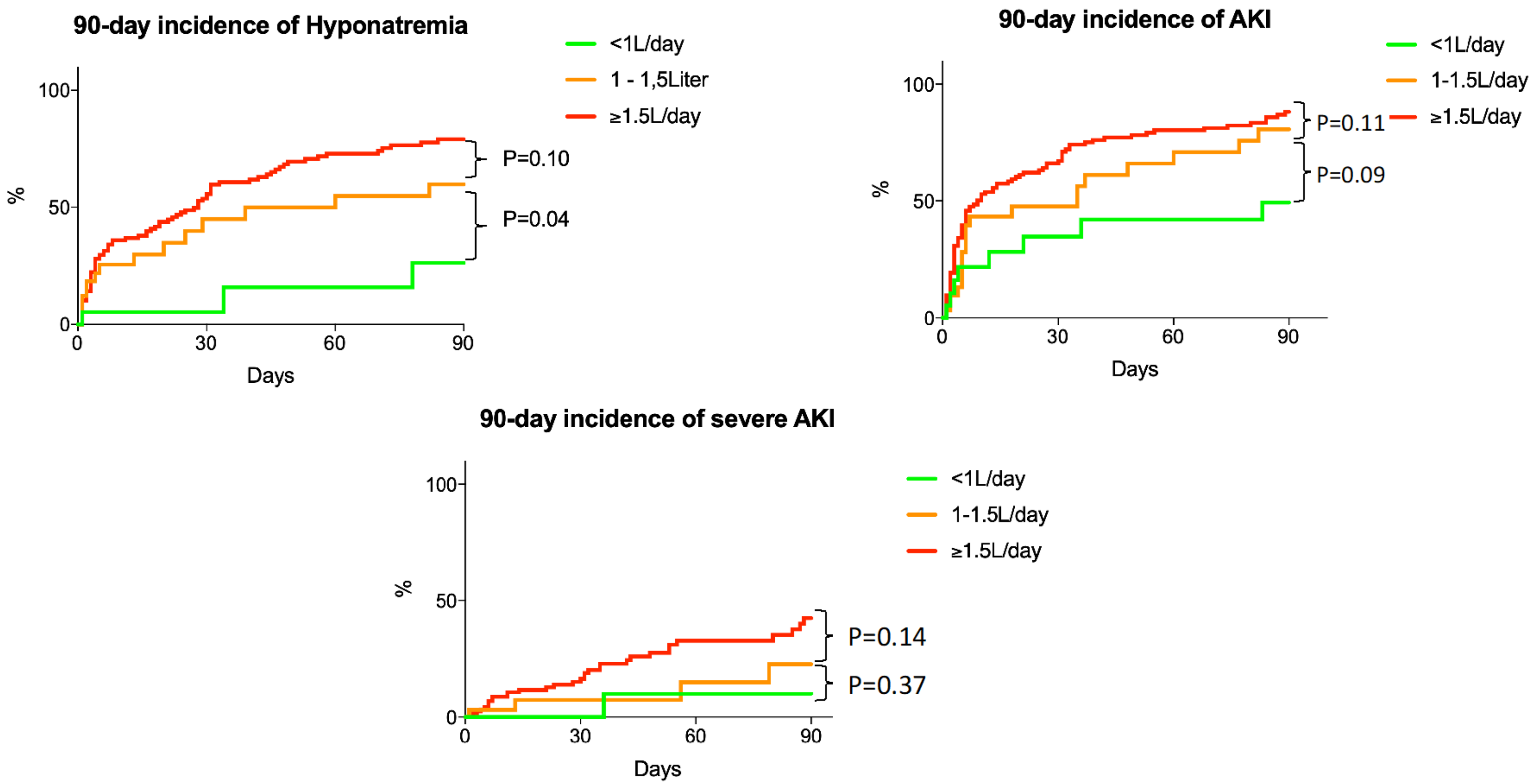

Supplementary Figure 2: Incidence of clinical complications stratified according to the daily volume drained. P-values were obtained using log-rank testing.

© 2023 Tergast TL et al. *JAMA Network Open.*

eFigure 3. ROC Curve

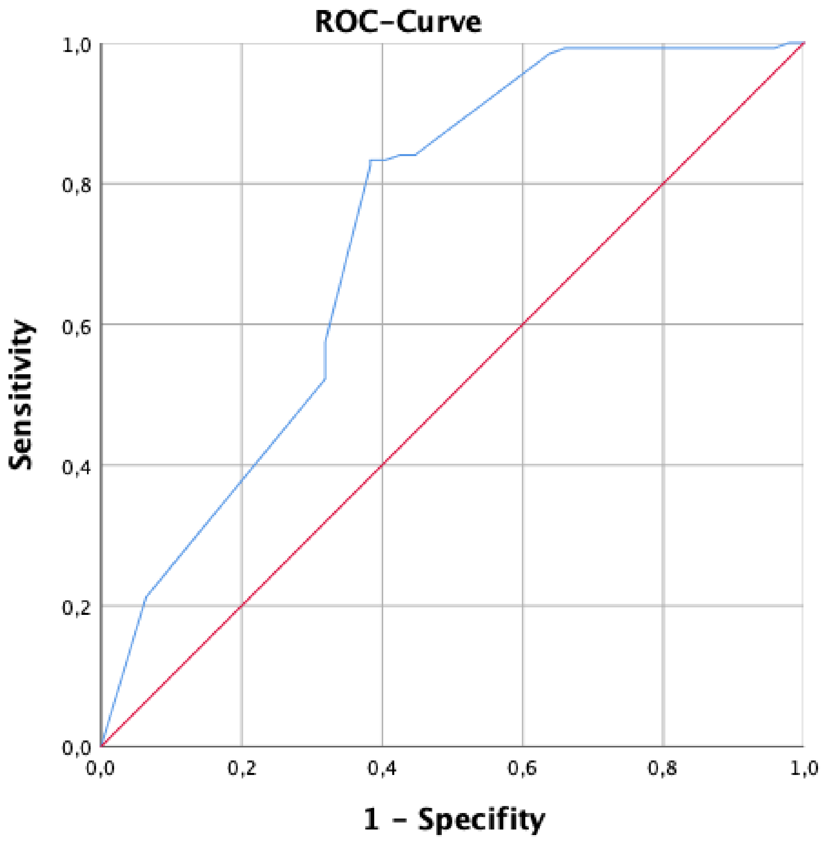

ROC curve, AUC=0.72. Patients without a clinical event that were censored within the first 90-days were excluded from this analysis. If patients that were censored in this analysis were included, the same cut-off of 1.5L/day was still optimal to predict clinical complications like AKI or hyponatremia. The optimal cut-off to predict only Hyponatremia was 1.45L/day and the optimal cut-off to predict only AKI was 1.6L/day.

© 2023 Tergast TL et al. *JAMA Network Open.*

eFigure 4. Ninety-Day Liver Transplantation-Free Survival and Rehospitalization Rate

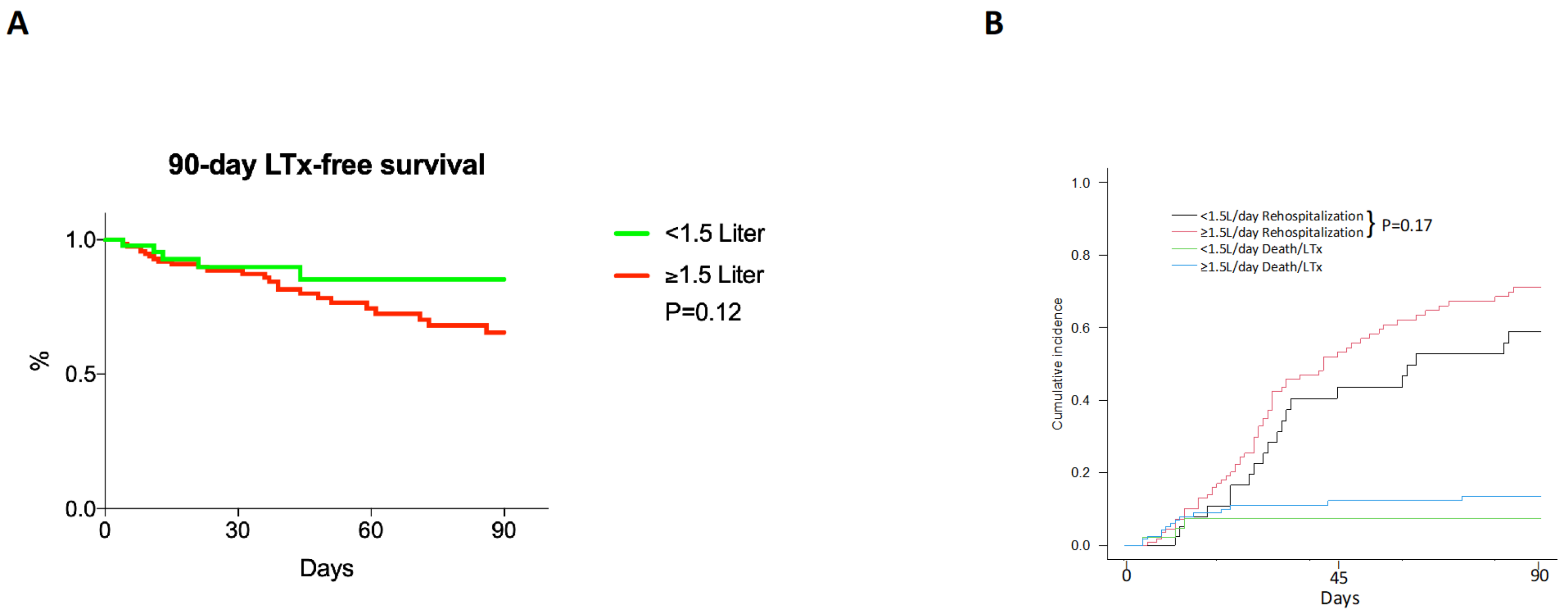

90-day LT-free survival (≥1.5L/day 25 events vs. <1.5L/day 5 events, A) and 90-day rehospitalization rate ((≥1.5L/day 65 events vs. <1.5L/day 20 events, B). The attached P-value for 90-day LTx free survival was calculated using log-rank testing. The P-value for 90-day rehospitalization rate was obtained using univariate competing risk analysis. Schoenfeld residual plots indicated that the proportional hazards assumption was met in the respective analyses.

© 2023 Tergast TL et al. *JAMA Network Open.*

eFigure 5. Serum Sodium, Serum Albumin Values, and Leukocyte Count Within 28-Day Follow Up

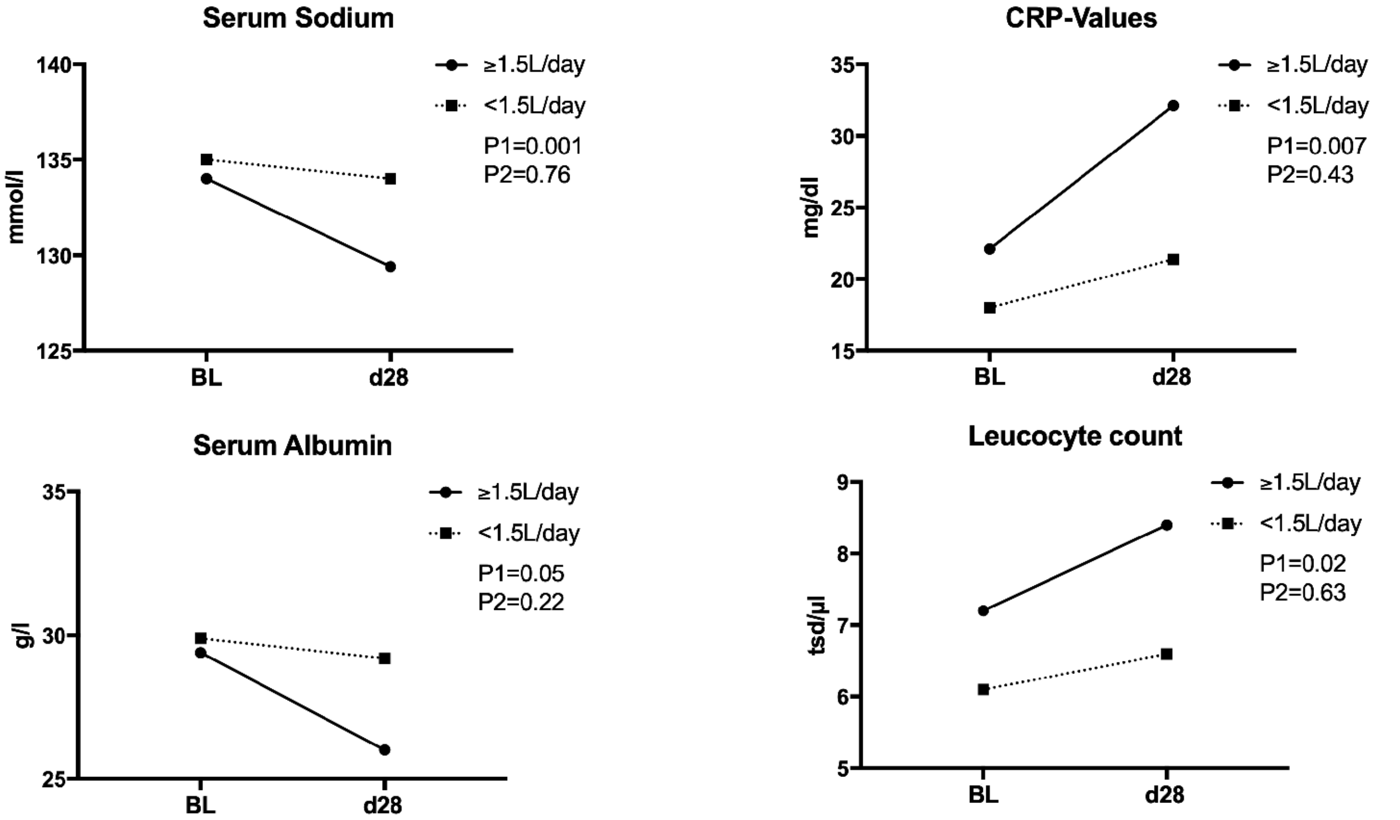

Serum sodium, serum albumin values and leukocyte count within 28-day follow-up in patients with drainage of either ≥1.5L/day and <1.5L/day. Attached p-values were calculated by paired t-testing. Binary logistic regression models adjusting for MELD, Age, platelet count and intake of NSBB to test whether an increase (for CRP and Leucocyte count) or decrease (for Sodium and Albumin) was independently associated with a drainage of ≥1.5L/day. Here, drainage of ≥1.5L/day was associated with an increase of inflammation parameters (CRP: HR: 2.67, P=0.01, Leucocyte count: HR: 2.01, P=0.002). Additionally, drainage of ≥1.5L/day was associated with a decrease of Serum Sodium (HR: 2.11, P=0.006) and there was a trend towards a decrease of Serum Albumin (HR: 1.99, P=0.08).

© 2023 Tergast TL et al. *JAMA Network Open.*

eFigure 6. Ninety-Day Incidences of Hyponatremia, AKI, and Severe AKI in Patients With Alfapump or PeCa

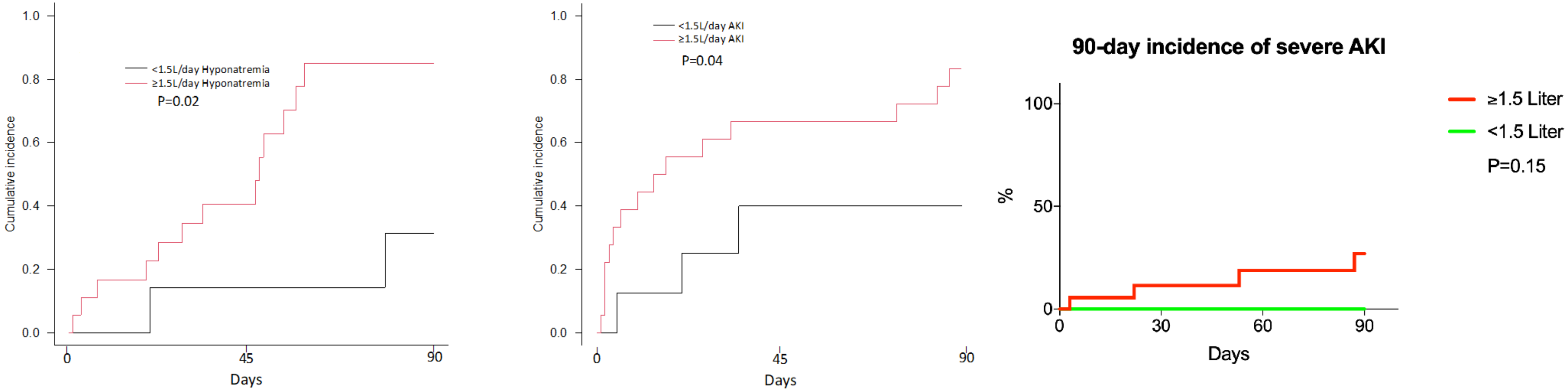

90-day incidences of hyponatremia (≥1.5L/day 13 events vs. <1.5L/day 2 events, A), AKI (≥1.5L/day 15 events vs. <1.5L/day 3 events B) and severe AKI (≥1.5L/day 4 events vs. <1.5L/day 0 events, C) of patients with daily taps of either ≥1.5L/day or <1.5L/day in patients with Alfapump. Univariate competing risk analysis was used for (A) and (B). Log-rank test was applied for (C), since there were no events in the <1.5L/day group. Schoenfeld residual plots indicated that the proportional hazards assumption was met in the respective analyses.

© 2023 Tergast TL et al. *JAMA Network Open.*

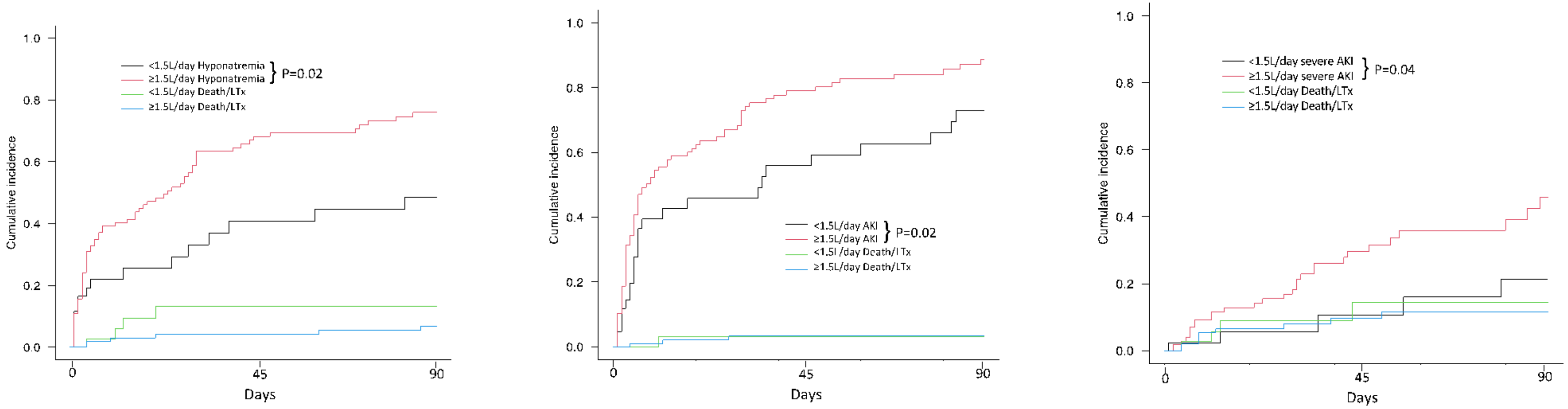

90-day incidences of hyponatremia (≥1.5L/day 72events vs. <1.5L/day 16 events, A), AKI (≥1.5L/day 83events vs. <1.5L/day 25 events, B) and severe AKI ((≥1.5L/day 28events vs. <1.5L/day 5 events, C) of patients with daily taps of either ≥1.5L/day or <1.5L/day in patients with PeCa. Univariate competing risk analysis was used to obtain the attached P-values. Schoenfeld residual plots indicated that the proportional hazards assumption was met in the respective analyses.

© 2023 Tergast TL et al. *JAMA Network Open.*

eFigure 7. Rehospitalization Rate in Patients With Either SOC or Daily Drainage of 1.5 L or More

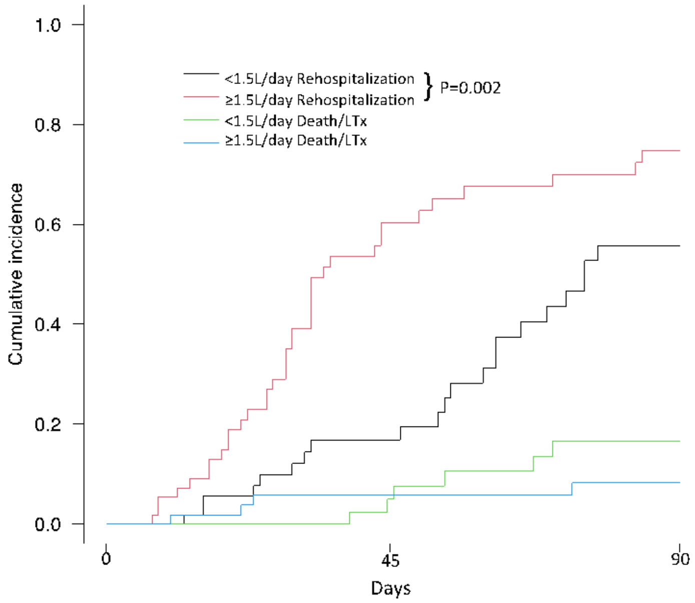

Supplementary Figure 7: Rehospitalization rate in patients with either SOC (21 events) or daily drainage of ≥1.5L/day (36 events). The respective Schoenfeld residual plot indicated that the proportional hazards assumption was met in the respective analyses.

Supplementary Figure 8

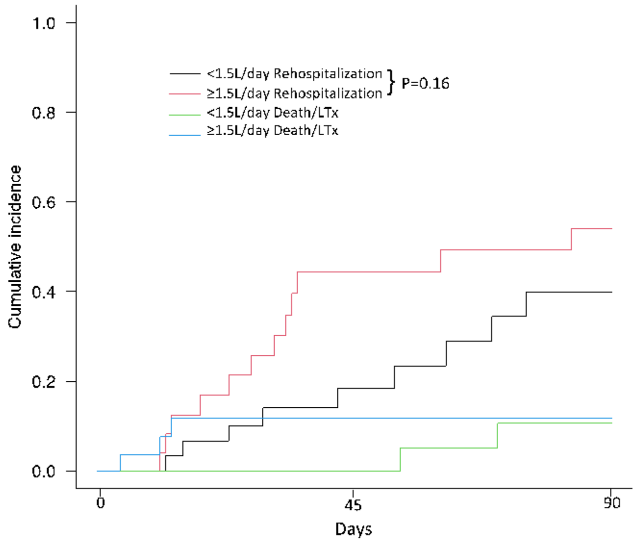

Supplementary Figure 8: Rehospitalization rate in patients with either SOC (n=9) or daily drainage of <1.5L/day (n=12). The attached P-values was obtained using univariate competing risk analysis. The respective Schoenfeld residual plot indicated that the proportional hazards assumption was met in the respective analyses.

© 2023 Tergast TL et al. *JAMA Network Open.*

eTable 1. Results of Multivariable Competing Risk Analysis

Parameter	HR	95% CI	P=
**Hyponatremia**			
**Age, per year**	1.00	0.98-1.03	0.71
**MELD, per point**	1.00	0.95 – 1.05	0.92
**Platelet count, per 10^5/µl**	1.02	0.77 – 1.0	0.76
**NSBB, yes**	1.31	0.89 – 1.94	0.17
**Leucocyte count, per 10^4/µl**	1.34	0.79 – 2.73	0.28
**Drainage volume >1.5L/day**	2.17	1.24 – 3.78	*0.006*
**AKI**			
**Age, per year**	1.00	0.98 – 1.02	0.68
**MELD, per point**	1.05	1.02 – 1.08	*0.001*
**Platelet count, per 10^5/µl**	0.83	0.61 – 1.14	0.32
**NSBB, yes**	0.88	0.59 – 1.32	0.55
**Leucocyte count, per 10^4/µl**	1.29	0.95 – 1.77	0.10
**Drainage volume >1.5L/day**	1.43	1.01 – 2.16	*0.04*
**Severe AKI**			
**Age, per year**	1.00	0.96 – 1.04	0.96
**MELD, per point**	1.06	0.97 – 1.14	0.16
**Platelet count, per 10^5/µl**	0.81	0.35 – 1.86	0.60
**NSBB, yes**	0.79	0.35 – 1.76	0.56
**Leucocyte count, per 10^4/µl**	1.69	0.78 – 4.09	0.60
**Drainage volume >1.5L/day**	2.65	1.21 – 6.54	*0.03*

© 2023 Tergast TL et al. *JAMA Network Open.*

Parameter	Std. Mean Difference - before matching	Std. Mean Differences - after matching
**Age, per year**	0.41	- 0.003
**MELD, per point**	0.66	- 0.06
**Platelet count, per 10^3/µl**	- 0.36	0.02
**NSBB, yes**	- 0.41	- 0.20
**Leucocyte count, per 10^3/µl**	- 0.28	- 0.01

eTable 2. Standardized Mean Differences Before and After PSM in Patients With Daily Drainage of 1.5 L/d or More Compared With Patients With SOC

Parameter	Std. Mean Difference - before matching	Std. Mean Differences - after matching
**Age, per year**	0.63	- 0.05
**MELD, per point**	- 0.11	- 0.10
**Platelet count, per 10^3/µl**	- 0.45	- 0.20
**NSBB, yes**	- 0.32	- 0.06
**Leucocyte count, per 10^3/µl**	- 0.46	- 0.15

eTable 3. Standardized Mean Differences Before and After PSM in Patients With Daily Drainage of Less Than 1.5 L/d Compared With Patients With SOC

© 2023 Tergast TL et al. *JAMA Network Open.*
