# Supplementary material for: Daily Low-Volume Paracentesis and Clinical Complications in Patients With Refractory Ascites
Source: JAMA Netw Open. 2023 Jul 6;6(7):e2322048. doi: 10.1001/jamanetworkopen.2023.22048 (PMC10326647; doi:10.1001/jamanetworkopen.2023.22048)
Supplement: Supplement 2. — Data Sharing Statement [file jamanetwopen-e2322048-s002.pdf]

Data Sharing Statement

Tergast. Daily Low-Volume Paracentesis and Clinical Complications in Patients With Refractory Ascites. *JAMA Netw Open*. Published July 06, 2023. doi:10.1001/jamanetworkopen.2023.22048

Data
Data available: No

Additional Information
Explanation for why data not available: Data is available only on request due to patient data protection policy.
